# Clinical efficacy of tetrandrine in artificial stone-associated silicosis: A retrospective cohort study

**DOI:** 10.3389/fmed.2023.1107967

**Published:** 2023-02-17

**Authors:** Wen-hong Wu, Yong-hong Feng, Chun-yan Min, Shao-wei Zhou, Zi-dan Chen, Li-min Huang, Wen-lan Yang, Guang-hong Yang, Jun Li, Jin Shi, Hua Quan, Ling Mao

**Affiliations:** ^1^Department of Pneumoconiosis, Shanghai Pulmonary Hospital, Tongji University, Shanghai, China; ^2^Shanghai Key Laboratory of Tuberculosis, Shanghai Pulmonary Hospital, Tongji University, Shanghai, China; ^3^School of Public Heath, The Key Laboratory of Environmental Pollution Monitoring and Disease Control, Ministry of Education, Guizhou Medical University, Guiyang, China; ^4^The Fifth People’s Hospital of Suzhou, Suzhou, China; ^5^Department of Pulmonary Function Test, Shanghai Pulmonary Hospital, Tongji University, Shanghai, China; ^6^Department of Health, Qingyang District Center for Disease Control and Prevention, Chengdu, China

**Keywords:** anti-fibrotic agents, artificial stone-associated silicosis, accelerated silicosis, tetrandrine, HRCT

## Abstract

**Background:**

Outbreaks of silicosis have occurred among workers in the artificial stone (AS) industry, and there is currently no effective antifibrosis treatment for silicosis.

**Design:**

A retrospective cohort study.

**Methods:**

We retrospectively analyzed the clinical data of 89 artificial stone-associated silicosis patients treated in Shanghai Pulmonary Hospital (China). Patients who agreed to be administered tetrandrine entered the observation group and those who disagreed entered the control group. Changes in chest HRCT, pulmonary function, and clinical symptoms of patients in two groups were compared pre- and post-treatment.

**Results:**

After treatment for 3–12 months, 56.5%–65.4% of patients in the observation group showed improvements in HRCT imaging, while there was no improvement in the control group (*p* < 0.05). Disease progression occurred in 0%–17.4% of patients in the observation group after 3–12 months of treatment compared with 44.4%–92.0% of patients in the control group (*p* < 0.05). After 3 months of treatment, the forced vital capacity (FVC), forced expiratory volume in 1 s (FEV_1_), and diffusing capacity of the lung for carbon monoxide (DLco) in the observation group increased by 136.7 ± 189.2 mL (*p* < 0.05), 124.2 ± 169.9 mL (*p* < 0.05), and 1.4 ± 2.3 mL/min/mmHg (*p* > 0.05), respectively, while those in the control group decreased (145.8 ± 356.5; 107.5 ± 272.1; 1.9 ± 3.8). After 6 months of treatment, FVC, FEV_1_, and DLco in the observation group increased by 207.8 ± 372.2 mL (*p* > 0.05), 107.8 ± 295.2 mL (*p* > 0.05) and 0.7 ± 6.0 mL/min/mmHg (*p* > 0.05), respectively, while those of the control group decreased (383.3 ± 536.7; 215.6 ± 228.9; 1.4 ± 1.7). The incidences of clinical symptoms such as cough, expectoration, dyspnea, chest tightness, and chest pain in the observation group were decreased-after treatment (all *p* < 0.05), while the incidences of these symptoms increased in the control group, although the change was not statistically significant (all *p* > 0.05).

**Conclusion:**

Tetrandrine can control and delay the progression of AS-associated silicosis fibrosis, with improved chest HRCT imaging and pulmonary function.

## Introduction

Artificial stone (AS) has been widely used in the manufacture of kitchen and bathroom countertops since the 1980s (1). The popularity of this product has increased all over the world due to its compact structure, low water absorption, high temperature resistance, and corrosion resistance. The first case report of AS-associated silicosis was published in Spain in 2010 (2). Since then, outbreaks of silicosis among AS workers have been reported around the world (3–5). It has been noted that AS-associated silicosis progresses more rapidly and has a worse prognosis than non-AS-associated silicosis due to exposing dust containing crystalline silica over 90% (6–8). Importantly, there is currently no effective antifibrosis treatment for silicosis.

Tetrandrine (Tet) is a bisbenzylisoquinoline alkaloid isolated from the root of *Stephania tetrandra* (9). Although the treatment effect of this compound on silicosis has been studied since the 1980s, its effect on the progression of silicosis fibrosis, especially in accelerated silicosis, remains to be established. Most previous studies about the effect of Tet on silicosis have focused on pulmonary function and clinical symptoms (10–12), while the manifestations of the condition in computed tomography imaging, a method used routinely for evaluation of lung disease, are still unclear. Therefore, in this study, we retrospectively analyzed the clinical data of AS-associated silicosis patients who had been treated with or without Tet in Shanghai Pulmonary Hospital (China) in recent years and focused on the high-resolution computed tomography (HRCT) findings.

## Materials and methods

### Study participants

In this retrospective cohort study, we analyzed the clinical data of patients with AS-associated silicosis who were treated in Pneumoconiosis Department of Shanghai Pulmonary Hospital between December 2015 and December 2021. Patients who agreed to be prescribed Tet entered the observation group and those who disagreed entered the control group.

### Eligibility

**Figure 1:**
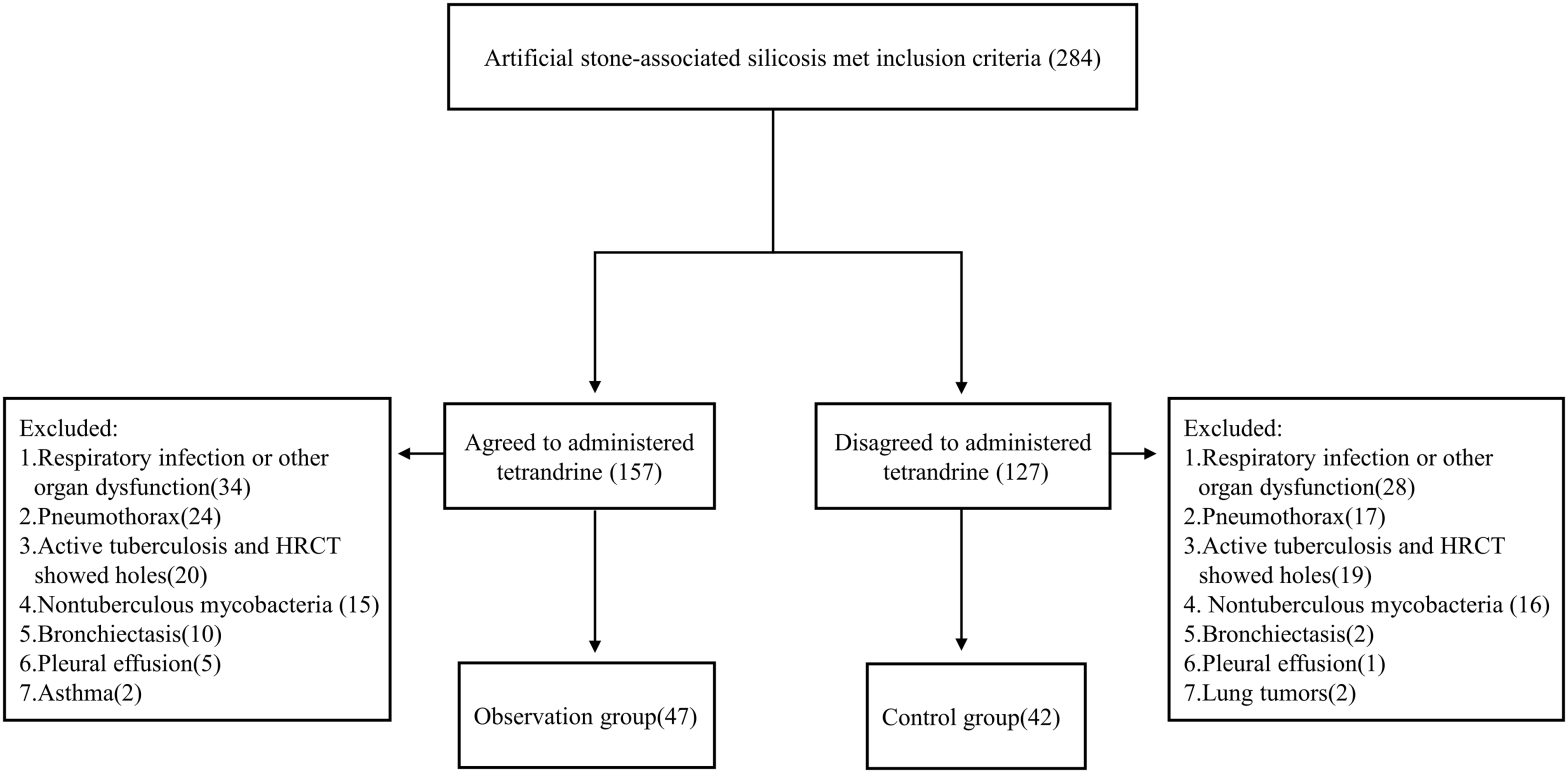
Study Flowchart of Patient Enrollment.

Patients were enrolled to this study according to the following inclusion criteria: (1) aged between 18 and 70 years; (2) exposed to AS dust while cutting, grinding, and drilling AS slabs before visiting hospital; (3) diagnosed as silicosis; (4) removed from continued dust exposure; and (5) had more than one chest HRCT examination performed. A total of 284 patients met the inclusion criteria. Patients with comorbidities such as tuberculous mycobacterial infection, lung tumor, respiratory infection, pneumothorax, pleural effusion, and asthma were excluded. Patients with other interstitial lung diseases or those with heart, brain, liver, kidney, and other organ dysfunction were also excluded. After exclusion, 89 patients were left, in which 47 patients were of observation group and 42 of control group (Figure 1).

### Therapeutic method

The patients in the control group were given symptomatic treatments as needed such as inhaled or oral bronchodilators, mucolytics as Mytol Standardized. Besides symptomatic treatment, the patients in the observation group received Tet (Zhejiang Jinhua Conba Biopharma Co., Ltd.; SFDA approval no. H33022075) administered at 60 mg/dose, three times per day for 6 days and then stopped for 1 day; this course of treatment lasted 3 months. After stopping for 1 month, the next course of treatment was administered.

### End-point measures

The primary end-point was the change in HRCT after treatment determined by re-reading and recording of images obtained pre-treatment, compared with those obtained at 3 months (after 2–4 months of treatment), 6 months (after 5–7 months of treatment), and 12 months (after 10–14 months of treatment) by a radiologist and a qualified physician from the pneumoconiosis department. The following indicators were recorded: ground-glass opacity (GGO), nodule opacity, progressive massive fibrosis (PMF), patchy opacity, emphysema, bullae, and dot-line opacity (13). Any of the following identified after treatment were classified as HRCT progression: (1) diffuse GGOs appear or increase; (2) PMF appear or extend; (3) diffuse nodules opacities increase; (4) emphysema and bullae appear or worsen. Any of the following identified after treatment were classified as HRCT improvement: (1) diffuse GGOs decrease or resolve; (2) smaller PMF without perifocal emphysema or worsening bullae; (3) diffuse nodules reduction; and (4) patchy opacities reduction. Stable HRCT was defined as no progression or improvement after treatment.

Secondary end-points were pulmonary function and clinical symptoms. Pulmonary function tests were performed as per the ATS/ERS recommendations (14, 15) and measured with a clinical spirometer (Jaeger Crop., Höchberg, Germany) by specialists from the department of pulmonary function in Shanghai Pulmonary Hospital. The pulmonary function indexes pre-treatment and at 3 months (after 2–4 months of treatment), 6 months (after 5–7 months of treatment), and 12 months (after 10–14 months of treatment) were obtained from the patients’ medical records. The following pulmonary function indexes were recorded: forced vital capacity (FVC) and its percentage of the predicted value (FVC%); forced expiratory volume in 1 s (FEV_1_) and its percentage of the predicted value (FEV_1_%); and diffusing capacity of the lung for carbon monoxide (DLco) and its percentage of the predicted value (DLco%).

The occurrence of clinical symptoms (cough, expectoration, dyspnea, chest tightness, and chest pain) in the two groups pre-treatment and at 3 months (after 2–4 months of treatment), 6 months (after 5–7 months of treatment), and 12 months (after 10–14 months of treatment) was recorded from the medical records.

Adverse effects: Adverse effects related to Tet treatment in the observation group were recorded according to outpatient and inpatient medical records.

### Statistical analysis

Quantitative data that were consistent with normal distribution were expressed as the mean ± standard deviation (SD), and the differences between groups were evaluated by Student’s *t*-test. When the variance was uneven, the corrected *t*-test was used. Quantitative data that were not consistent with normal distribution were expressed as the median and interquartile range, and the differences between groups were evaluated by the Mann–Whitney *U*-test. Qualitative data were expressed as relative numbers, and the Chi-square test or Fisher’s exact test was used for comparison between groups. The Kaplan–Meier method was used to estimate the rate of progression, and the log-rank test was used to compare the distribution of survival curves between two groups. All data analyses were conducted using SPSS version 25.0, Microsoft Excel 2021 for database construction, and GraphPad Prism 8.3.0 for drawing. *p* < 0.05 was set as the threshold for statistical significance. It should be noted that indications of pulmonary function were not consistent with normal distribution, non-parametric tests were adopted for the statistical significance of the differences. To facilitate intuitive interpretation of the data, normally distributed data were presented.

## Results

### Demographic characteristics

A total of 89 patients were enrolled in this study consisting of 47 cases in the observation group (average age 37.4 ± 12.1 years) and 42 cases in the control group (average age 37.7 ± 8.6 years). There were no significant differences in age, sex, body mass index (BMI), dust-exposure time, smoking status, or PMF rates between the two groups (all *p* > 0.05; Table 1). The shortest dust-exposure time among all subjects was 2 years and the longest was 20 years.

**Table 1:** Demographic characteristics of the observation and control groups.

Characteristics	Observation (*n* = 47)	Control (*n* = 42)	*χ*^2^/*t*	Value of *p*
Male, *n* (%)	47 (100.0)	41 (97.6)	0.003	0.955
Age at treatment (years)	37.4 ± 12.1	37.7 ± 8.6	0.095	0.924
Age at onset of dust exposure (years)	29.4 ± 10.6	28.4 ± 7.4	0.540	0.591
BMI (kg/m^2^)	24.1 ± 3.3	22.9 ± 3.5	1.524	0.132
Dust-exposure time (years)	6.4 ± 3.6	6.9 ± 3.6	0.582	0.562
Current/former smoker, *n* (%)^a^	27 (57.5)	22 (52.4)	0.230	0.632
PMF, *n* (%)	16 (34.0)	8 (19.1)	2.532	0.112

BMI, body mass index; PMF, progressive massive fibrosis.

a Patients who had quit smoking 1–10 years before the first registration were classified as former smokers, and those who had quit for more than 10 years were classified as never smokers.

### Case examples

**Figure 2:**
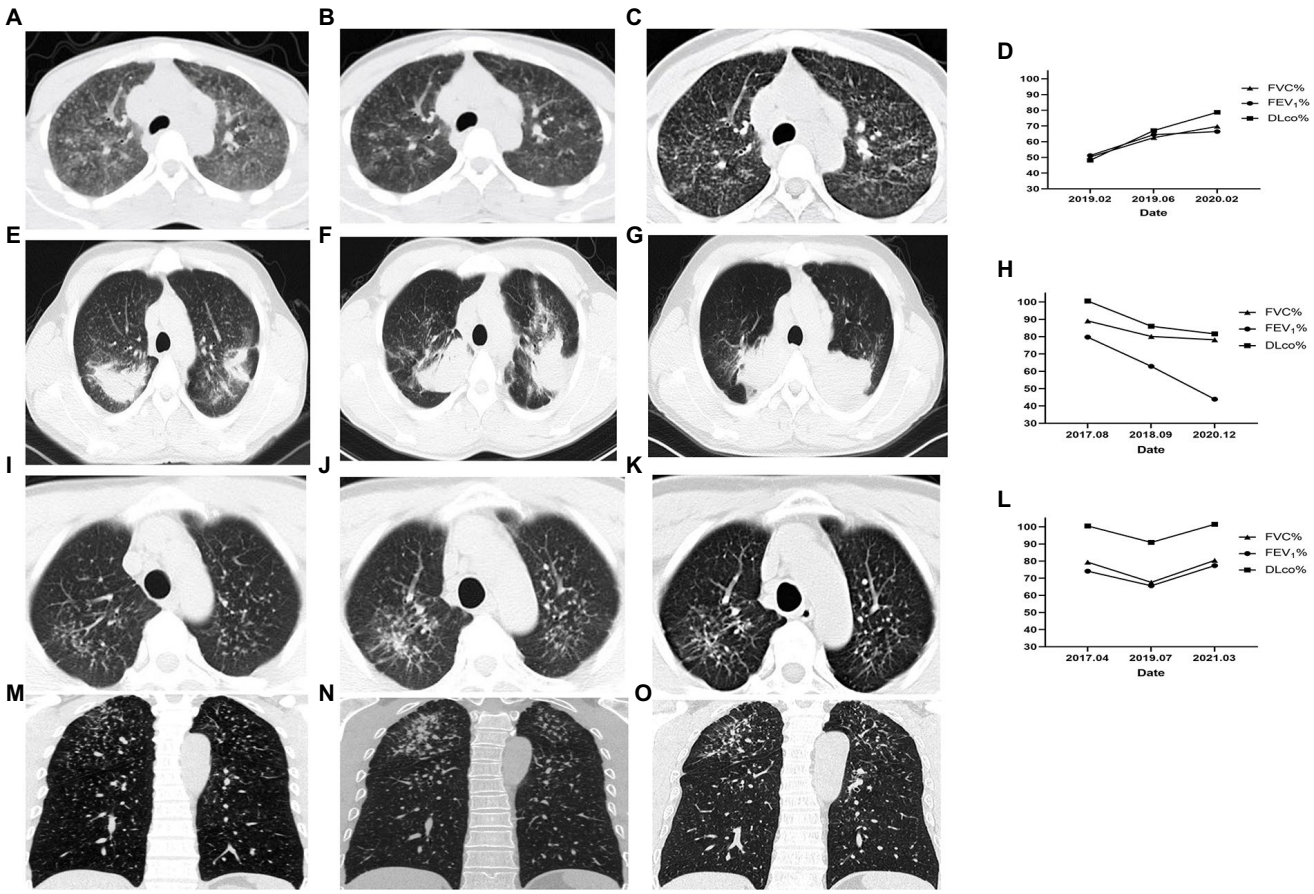
Changes of HRCT and pulmonary function of representative cases. Case 1 of the observation group. **(A)** HRCT showed diffuse GGOs, extensive nodular opacities, and para-aortic lymph node enlargement at baseline. **(B)** GGOs was significantly reduced after 4 months. **(C)** GGOs dissipated without coalescence or PMF emerging after 31 months of treatment. **(D)** The pulmonary function indexes FVC%, FEV_1_%, and DLco% showed an increasing trend. Case 2 of the control group. **(E)** HRCT showed PMF on bilateral lungs at baseline. **(F)** The left lung PMF enlarged and the right lung PMF contracted centripetally with emphysema developing 13 months later. **(G)** The lesion on HRCT continued to progress and the emphysema worsened. **(H)** Correspondingly, the pulmonary function gradually decreased. Case 3 began Tet treatment after the second HRCT. **(I,M)** Cross-sectional and coronal HRCT images showed diffusely distributed nodules at baseline. **(J,N)** Nodule opacities increased and coalescence emerged in the right upper lung 27 months later. **(K,O)** GGO around the coalescence in the right upper lung dissipated and the coalescence had not progressed after 20 months of Tet treatment. **(L)** The pulmonary function decreased over 27 months without Tet treatment and increased over 20 months with Tet treatment. This case was included in the observation group.

In Case 1, a 26-year-old male in observation group with 5 years of exposure, the manifestation observed in the HRCT images did not show deterioration over 31 months and pulmonary function improved. In Case 2, a 29-year-old male in the control group with 6 years of exposure, aggravated on HRCT and pulmonary function over 32 months. In Case 3, a 44-year-old male in observation group with 8 years of exposure, HRCT progressed and pulmonary function decreased in 27 months after first registration and improved after 20 months’ treatment with Tet (Figure 2).

### Changes in HRCT

The chest HRCT characters of AS-associated silicosis were GGO, nodule opacities, patchy opacity, PMF, emphysema, bullae, and dot-line opacity. There were no significant differences between the observation and control groups in the incidence of changes in GGO, nodule opacities, patchy opacity, PMF, emphysema, bullae, and dot-line opacity at baseline (all *p* > 0.05).

After 3, 6, and 12 months of treatment, the rates of improvement in the HRCT features in the observation group were 65.38, 56.52, and 63.16%, respectively, while no improvements were observed in the control group, with a statistically significant difference between the two groups (*p* < 0.001, Table 2). Improvements in HRCT imaging are mainly manifested by the reduction or dissipation of ground-glass opacities, the reduction of nodal opacities, and the shrinkage of PMF. The rates of progression rates in the control group at 3, 6, 12 months were 52.94, 44.44, and 92.00%, while the rates in the observation were 0, 17.39 and 5.26%, respectively, with a statistically significant difference between the two groups (*p* < 0.001, Table 2).

**Table 2:** Changes in HRCT features of the observation and control groups after treatment [*n* (%)].

Group	Changes	Observation	Control	Fisher	Value of *p*
3 M		***n* = 26**	***n* = 17**	28.419	<0.001
	Improved	17 (65.4)	0		
	Stable	9 (34.6)	8 (47.1)		
	Progressive	0	9 (52.9)		
6 M		***n* = 23**	***n* = 9**	9.476	0.007
	Improved	13 (56.5)	0		
	Stable	6 (26.1)	5 (55.6)		
	Progressive	4 (17.4)	4 (44.4)		
12 M		***n* = 19**	***n* = 25**	38.102	<0.001
	Improved	12 (63.2)	0		
	Stable	6 (31.6)	2 (8.0)		
	Progressive	1 (5.3)	23 (92.0)		

### Pulmonary function

Most of the subjects exhibited restrictive ventilatory dysfunction and impaired diffusion function at baseline. In the 3-month cohort, the FVC%, FEV_1_%, and DLco% values of the control group were all significantly higher than those of the observation group at enrollment (*p* < 0.05; Table 3). After 3 months of treatment, both FEV_1_% and DLco% values of the observation group increased, while those of the control group decreased, with statistically significant differences between the pre- and post-treatment values of these indexes between the two groups (*p* < 0.05; Table 3). In the 6-month cohort, the FVC% and FEV_1_% values of the observation group increased, while those of the control group decreased, with statistically significant differences between the pre- and post-treatment values of these indexes between the two groups (*p* < 0.05; Table 3).

**Table 3:** Changes of pulmonary function indexes of the observation and control groups after treatment.

Groups	Indicators	Time	Observation	Control	*t*	Value of *p*
3 M			***n* = 12**	***n* = 12**		
	FVC%	Before	62.7 ± 16.6	78.0 ± 10.1	2.729	0.012
		After	67.0 ± 18.3	76.1 ± 15.3	1.335	0.196
		Difference	4.3 ± 4.7	−1.9 ± 9.1	2.072	0.054
	FEV_1_%	Before	57.1 ± 17.2	76.2 ± 11.5	3.203	0.004
		After	61.6 ± 19.8	74.7 ± 16.3	1.767	0.091
		Difference	4.5 ± 5.4	−1.6 ± 7.8	2.214	0.037
	DLco%	Before	66.6 ± 19.8	83.6 ± 15.6	2.266	0.034
		After	74.4 ± 16.4	74.6 ± 19.3	0.022	0.983
		Difference	6.2 ± 10.5	−7.0 ± 15.7	2.33	0.030
6 M			***n* = 9**	***n* = 9**		
	FVC%	Before	64.3 ± 19.3	75.8 ± 18.2	1.306	0.210
		After	69.4 ± 23.7	68.0 ± 22.0	0.132	0.897
		Difference	5.2 ± 8.6	−7.8 ± 10.9	2.715	0.015
	FEV_1_%	Before	59.8 ± 19.3	68.3 ± 25.0	0.807	0.432
		After	62.9 ± 24.84	62.9 ± 28.7	0.003	0.998
		Difference	3.1 ± 8.2	−5.3 ± 5.8	2.538	0.022
	DLco%	Before	77.1 ± 16.8	74.0 ± 29.0	0.273	0.789
		After	79.0 ± 16.6	67.5 ± 28.1	1.05	0.309
		Difference	1.9 ± 26.5	−6.5 ± 5.6	0.928	0.367

**Figure 3:**
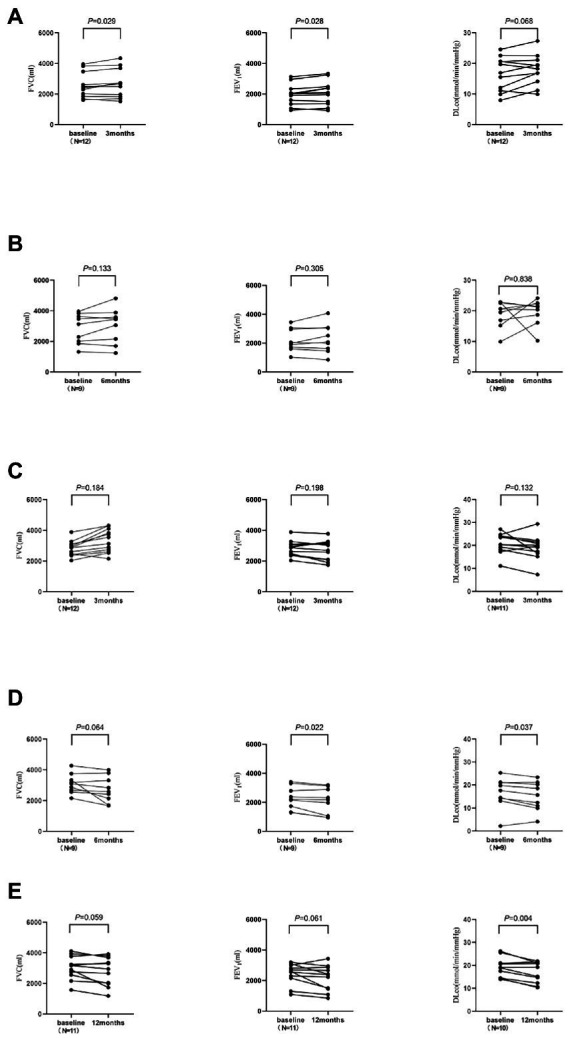
The changes of pulmonary function in the observation group and control group. DLco was not measured for one subject in each of the 3-month and 12-month cohorts. **(A)** The 3-month cohort of observation group. **(B)** The 6-month cohort of observation group. **(C)** The 3-month cohort of control group. **(D)** The 6-month cohort of control group. **(E)** The 12-month cohort of control group.

In the 3-and 6-month cohorts, the average increases of FVC in the observation group were 136.7 ± 189.2 mL (*p* = 0.029) and 207.8 ± 372.3 mL (*p* = 0.133), respectively, while the average increases in of FEV_1_ were 124.2 ± 169.9 mL (*p* = 0.028), 107.8 ± 295.2 mL (*p* = 0.305), respectively, and the averages increases of DLco were 1.4 ± 2.3 mL/min/mmHg (*p* = 0.068), 0.7 ± 6.0 mL/min/mmHg (*p* = 0.838), respectively (Figure 3). Pulmonary function improvement cases were accompanied by a decrease or disappearance of GGO on HRCT images.

In the 3-, 6-, and 12-month cohorts, the average decreases of FVC in the control group were 145.8 ± 356.5 mL, 383.3 ± 536.7 mL, and 250.0 ± 388.8 mL, respectively (all *p* > 0.05), while the average decreases in FEV_1_ were 107.5 ± 272.1 mL (*p* = 0.198), 215.6 ± 228.9 mL (*p* = 0.022), and 266.4 ± 419.3 mL (*p* = 0.061), respectively, and the average decreases in DLco were 1.9 ± 3.8 mL/min/mmHg (*p* = 0.132), 1.4 ± 1.7 mL/min/mmHg (*p* = 0.037), and 2.4 ± 2.0 mL/min/mmHg (*p* = 0.004; Figure 3).

### Cumulative progression rate

**Figure 4:**
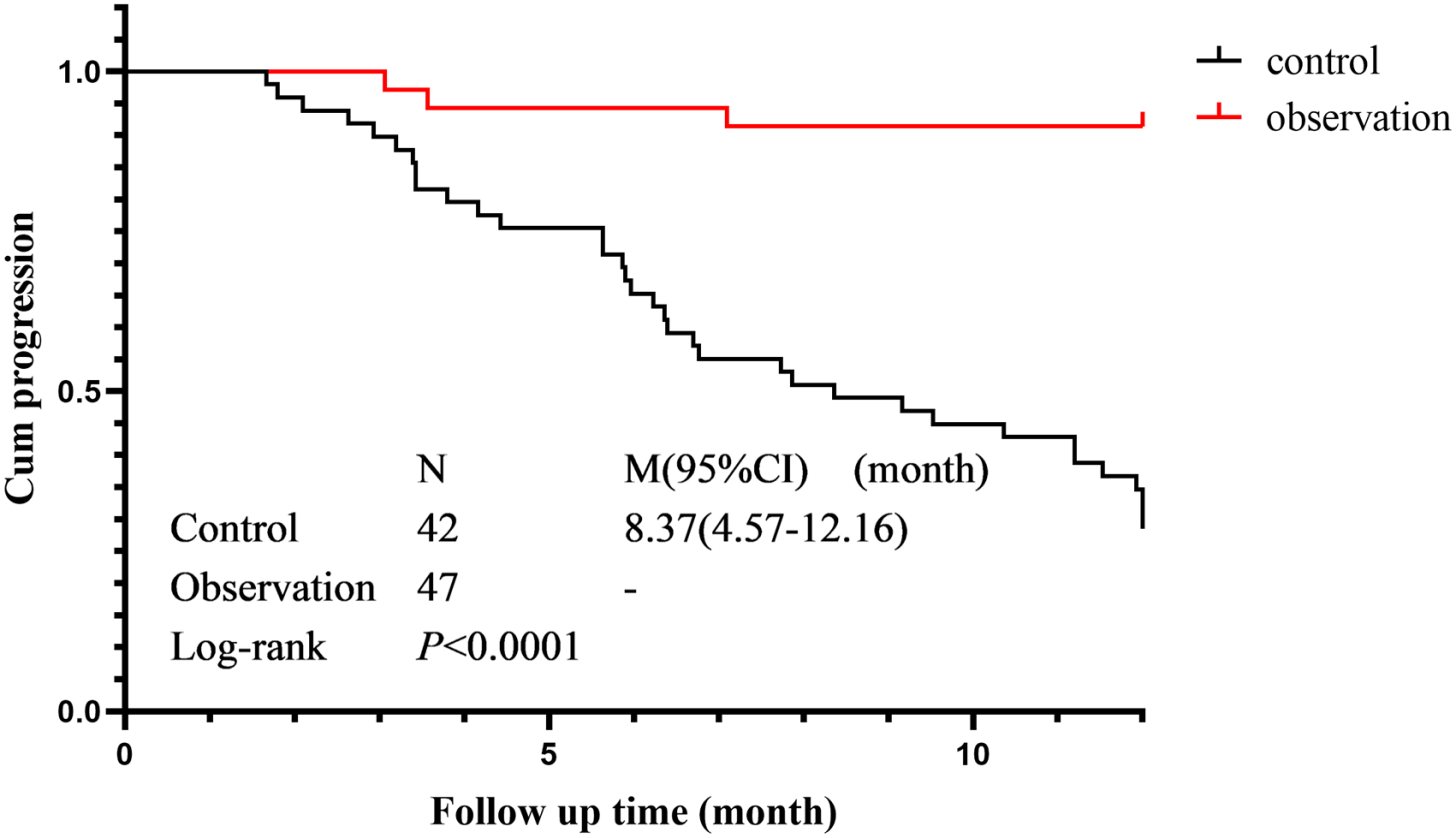
Cumulative progression rate in the observation group and control groups.

The cumulative progression curves of the two groups based on HRCT progression as the terminal event are shown in Figure 4. There was a statistically significant difference in the cumulative progression rate between the control group (median progression survival time 8.367 months, 95% CI 4.57–12.16) and the observation group (Log-Rank *p* < 0.001). The cumulative progression rate in the observation was significantly lower than that in the control group.

### Clinical symptoms

There were no significant differences in the incidence of clinical symptoms including cough, expectoration, dyspnea, chest tightness, and chest pain between the two groups at baseline (all *p* > 0.05). The incidence of all clinical symptoms in the observation group reduced in the 3-, 6-, and 12-month cohorts, with significant differences compared with those in the control group (all *p* < 0.05; Table 4).

**Table 4:** Change of clinical symptoms [*n* (%)].

Indicator	3 M			6 M			12 M		
	Observation (*n* = 42)	Control (*n* = 35)	Value of *p*	Observation (*n* = 25)	Control (*n* = 19)	Value of *p*	Observation (*n* = 22)	Control (*n* = 27)	Value of *p*
Cough									
Before	37 (88.1)	31 (88.6)	0.948	22 (88.0)	17 (89.5)	0.879	19 (86.4)	24 (88.9)	0.789
After	15 (35.7)	33 (94.3)	<0.001	11 (44.0)	19 (100.0)	<0.001	9 (40.9)	27 (100.0)	<0.001
Expectoration									
Before	25 (59.5)	24 (68.6)	0.411	15 (60.0)	13 (68.4)	0.565	12 (54.6)	19 (70.4)	0.253
After	19 (45.2)	27 (77.1)	0.004	11 (44,0)	15 (79.0)	0.020	9 (40.9)	22 (81.5)	0.003
Dyspnea									
Before	26 (61.9)	25 (71.4)	0.379	16 (64.0)	13 (68.4)	0.759	14 (63.6)	19 (70.4)	0.617
After	16 (38.1)	27 (77.1)	0.001	10 (40.0)	15 (79.0)	0.010	7 (31.8)	23 (85.2)	<0.001
Chest tightness									
Before	36 (85.7)	32 (91.4)	0.437	21 (84.0)	17 (89.5)	0.600	18 (81.8)	23 (85.2)	0.751
After	19 (45.2)	35 (100.0)	<0.001	12 (48.0)	19 (100.0)	<0.001	10 (45.5)	27 (100.0)	<0.001
Chest pain									
Before	24 (57.1)	15 (42.9)	0.212	14 (56.0)	8 (42.1)	0.361	13 (59.1)	16 (59.3)	0.990
After	10 (23.8)	17 (48.6)	0.023	6 (24.0)	11 (57.9)	0.022	6 (27.3)	17 (63.0)	0.013

### Adverse effects

The adverse effects related to Tet in the observation group included facial pigmentation (9/47, 19.2%), diarrhea (4/47, 8.5%), skin itching (2/47, 4.3%), nausea (1/47, 2.1%), fatigue (1/47, 2.1%), lethargy (1/47, 2.1%), and transient hepatic dysfunction (1/47, 2.1%).

## Discussion

Silicosis is a systemic disease characterized by pulmonary fibrosis, mainly caused by long-term inhalation of dust containing free silica. AS-associated silicosis is characterized by a shorter dust-exposure time, faster disease progression, higher lung transplantation rate, and mortality than natural stone silicosis (16). HRCT showed AS-associated silicosis with a 1-year progression rate of 72.2% (16). For a long time, there is no specific treatment for silicosis. Treatment of the comorbidities (such as tuberculosis) is the main treatment for silicosis, which is far from enough for the patients, especially for accelerated silicosis. It is necessary to take measures for controlling the progression of fibrosis so as to delay the decline of lung function and reduce premature deaths. Effective anti-fibrotic drugs are urgently required to treat accelerated silicosis with rapid progression and a higher mortality rate (16, 17). Drug screens for the treatment of silicosis were carried out in China since the 1970s, and tetrandrine was one of chemicals selected with potential effects.

Results from animal experiments indicated that Tet could promote the activity of superoxide dismutase (SOD) in lung tissue, inhibit the release of fibrotic factor from lung macrophages, and attenuate lung inflammation. Tet also inhibited the synthesis and release of glycosaminoglycans and lipids, inhibited the transcription of collagen genes, and degraded the collagen in the silicic nodules formed, thereby reducing and delaying silicosis fibrosis (18–20).

In our study, 56.5–65.4% of the patients in the observation group showed improvement in HRCT after 3–12 months of treatment. Cumulative progression rate analysis also showed that the progression rate of the observation group was significantly lower than that of the control group. The FVC, FEV1, and DLco values of the observation group improved with varying degrees after 3–6 months, while significant decreases in FEV_1_ and DLco were observed in the control group. Improvements in lung function were always accompanied by improvements in HRCT imaging. These results are consistent with the early clinical research reported in the 1990s, which showed that treatment with combination of Tet with quinolyl piperazine hydroxyl phosphate (QOHP) or poly-2-vinyl pyridine-nitrogen oxide (PVNO) resulted in obvious inhibition of the process of fibrosis and improvement of clinical symptoms. Accelerated silicosis with the combination treatment showed X-ray improvement rates (22.4%) higher than other silicosis patients (5.7%) (21). In another study, treatment with Tet for 6 months resulted in improvements on chest X-rays in 24.8% of 117 silicosis patients (22). The higher rate of improvement in HRCT features in the observation group of the current study may be due to the higher density resolution of this imaging technique compared with chest X-rays.

Some limitations of this study should be noted. As a retrospective study, there may be selective bias exists in the study subjects; and cases with insufficient lung function test information were also a problem. Furthermore, in this study, we collected clinical data during only 12 months of treatment, the long-term efficacy of Tet treatment in accelerated and chronic silicosis patients remains to be established. Even so, the striking results achieved by the study cannot be ignored. As artificial stone-associated silicosis is a type of progressive fibrosing interstitial lung diseases (PF-ILD), this study indicated that the potential effect of Tet in treatment of other PF-ILD is worthy of further investigation.

## Conclusion

Tet had a definite therapeutic effect on patients with accelerated silicosis with improvements in HRCT features and pulmonary function combined with delayed progression of fibrosis, few adverse effects were recorded.

## Data availability statement

The raw data supporting the conclusions of this article will be made available by the authors, without undue reservation.

## Ethics statement

The studies involving human participants were reviewed and approved by the Medical Ethics Committee of Shanghai Pulmonary Hospital. The patients/participants provided their written informed consent to participate in this study.

## Author contributions

W-hW, Y-hF, C-yM, and LM applied conception, designed the research, and wrote the article. W-hW, LM, L-mH, JS, and HQ collected the clinical data. W-lY and LM interpreted the lung function data. S-wZ and Z-dC interpreted the radiologic data. W-hW, L-mM, Y-hF, G-hY, and JL analyzed and interpreted the clinical data. Y-hF and LM provided financial support fund and conducted the entire research. All authors contributed to the article and approved the submitted version.

## Funding

This work was supported by National Natural Science Foundation of China (nos. 81771692 and 81971558).

## Conflict of interest

The authors declare that the research was conducted in the absence of any commercial or financial relationships that could be construed as a potential conflict of interest.

## Publisher’s note

All claims expressed in this article are solely those of the authors and do not necessarily represent those of their affiliated organizations, or those of the publisher, the editors and the reviewers. Any product that may be evaluated in this article, or claim that may be made by its manufacturer, is not guaranteed or endorsed by the publisher.
